# Effect of COVID-19 quarantine on cognitive, functional and neuropsychiatric symptoms in patients with mild cognitive impairment and dementia

**DOI:** 10.1007/s40520-022-02113-z

**Published:** 2022-03-24

**Authors:** Laura Vernuccio, Davide Sarà, Florenza Inzerillo, Giuseppina Catanese, Angela Catania, Miriam Vesco, Federica Cacioppo, Ligia J. Dominguez, Nicola Veronese, Mario Barbagallo

**Affiliations:** 1 Geriatric Unit, Azienda Ospedaliera Universitaria Policlinico Paolo Giaccone, Palermo, Italy; 2 Geriatric Unit, Department of Internal Medicine and Geriatrics, University of Palermo, Via del Vespro, 127, 90127 Palermo, Italy; 3 School of Medicine, “Kore” University of Enna, Enna, Italy

**Keywords:** Dementia, Mild cognitive impairment, COVID-19, Quarantine, Neuropsychiatric symptoms

## Abstract

**Background:**

During the last two years, COVID-19 affected older people with dementia or mild cognitive impairment (MCI), but conflicting and sparse results are still present. The objective of this study was to investigate the frequency and type of changes in functional, cognitive and behavioral and psychological symptoms of dementia (BPSD), and caregiver’s stress during the period of quarantine in 2020 in patients affected by dementia/MCI living in Palermo, Sicily.

**Methods:**

Outpatients affected by MCI/dementia were evaluated before and after COVID-19 quarantine. Functional status was investigated using basic and instrumental activities of daily living (ADL); cognitive performance with the mini-mental state examination; BPSD through the neuropsychiatric inventory (NPI). All scales were reported as pre/post-COVID-19 quarantine and a logistic regression analysis was performed for investigating the factors associated with worsening in NPI in patients and their caregivers.

**Results:**

One hundred patients (mean age 77.1; females = 59%) were evaluated over a median of 10 months. In the sample as whole, a significant decline in functional and cognitive status was observed (*p* < 0.001 for both comparisons). The NPI significantly increased by 3.56 ± 8.96 points after the COVID-19 quarantine (*p* < 0.0001), while the caregivers’ stress increased by 1.39 ± 3.46 points between the two evaluations (*p* < 0.0001). The decline was more evident in people with milder dementia. Higher values of instrumental ADL at baseline were associated with a significant lower worsening in NPI and caregiver’s stress.

**Conclusions:**

COVID-19 quarantine negatively affected functional, cognitive, and neuropsychiatric symptoms in older people affected by dementia/MCI, highlighting the impact of COVID-19 quarantine for this population.

## Introduction

An emerging genotype of coronavirus, severe acute respiratory syndrome coronavirus 2 (SARS-CoV-2), led to a new medical condition called Coronavirus Disease-19 (COVID-19) since the end of 2019 [1]. This virus arrived in Europe in January 2020, with the first positive patient identified in Germany [2]. COVID-19 is mainly a respiratory disease, but increasing literature has shown that neurological and cardiovascular complications are frequent [3, 4]. Older and frailer adults have the worse prognosis, and some authors have indicated that COVID-19 could be considered as a geriatric syndrome [5].

Government authorities have introduced mitigation strategies based on lockdown measures, travel restrictions, and mass quarantine in an attempt to containing and managing COVID-19. Confinement and isolation have been proven to be highly effective for the control of spreading COVID-19 epidemic initially and pandemic thereafter. However, previous outbreaks of SARS and Middle East respiratory syndrome (MERS) showed that quarantine has negative effects on mental health, with increased psychiatric symptoms particularly related to stress reactions such as anxiety, depression, and anguish [6]. Considering findings from previous literature and preliminary observations during the COVID-19 pandemic, the scientific community has launched an alarm about a possible imminent “pandemic” of psychiatric disorders, also in older people [7].

Similar complications could be exponentially present in persons affected by dementia, who are often frail and depend on caregivers for their activities of daily living (ADL). Moreover, they often need support from a network of social and health services resources. In this already restricted lifestyle, extended lockdown with imposed self-isolation and modification or deprivation of usual daily activities may represent a further relevant stressor in persons with cognitive decline and dementia [8]. Caregivers are as well at high risk of mood disorders, such as anxiety and depression, due to changes in their daily routines while helping their loved ones [8].

A call of action for a plan to evaluate and counteract mental illnesses in the COVID-19 post-pandemic phase has been launched for the general population. However, knowledge on the cognitive effects of lockdown and relative restrictions in patients with dementia and mild cognitive impairment (MCI) is still limited and reports have shown conflicting results [9–12].

Since the results regarding the impact of quarantine and COVID-19 in people with dementia and MCI are still not univocal, the aim of the present study was to evaluate the frequency and type of functional and cognitive changes, as well as behavioral, and psychological symptoms of dementia (BPSD) and caregiver’s stress during the period of quarantine in 2020 in patients affected by dementia and MCI living in Palermo, Sicily, Italy.

## Methods

### Participants

All outpatients undergoing evaluation at the Geriatric Unit’s Cognitive Disorders and Dementia Center of the University Hospital “Azienda Ospedaliera Universitaria Policlinico Paolo Giaccone” from Palermo, Italy, were consecutively enrolled in the study.

Inclusion criteria were: (1) available comprehensive evaluation before and after COVID-19 pandemic lockdown; (2) age ≥ 60 years; (3) diagnosis of MCI or dementia using the Diagnostic and Statistical Manual of Mental Disorders (DSM)-V criteria. We excluded from the analyses those patients who did not have a complete evaluation regarding functional and cognitive performance, as well as those with a primary psychiatric disorder such as bipolar disorder or schizophrenia.

All analyzed data were collected as part of the routine clinical diagnostic and treatment procedures, following the Declaration of Helsinki’s Ethical Principles for Medical Research involving human subjects. In agreement with the current Italian law, we informed our local Ethical Committee about the present observational research regarding normal critical practice by sending a formal letter.

### General information

During the first visit, we collected information on age, gender, living conditions (categorized as in family vs. nursing home vs. alone), and marital status (married vs. other options). Information regarding medications were recorded using medical records, interviews with the caregivers, or phone calls with the general practitioners. This included the most common medications used by the participants for the treatment of dementia and neuropsychiatric symptoms (i.e., antipsychotics, mood stabilizers, anti-Parkinson medications, benzodiazepines, antidepressants, and hypnotics).

### Diagnosis of dementia and mild cognitive impairment

The diagnosis of dementia and MCI was formulated according to validated criteria, by means of the anamnesis, physical examination, imaging evaluation, and medical documentation. Based on this information, the diagnosis of dementia or MCI was made according to the diagnostic criteria proposed by the DSM-V [13, 14].

According to the DSM-V, dementia was also categorized in Alzheimer’s disease (AD), vascular dementia, mixed, secondary to Parkinson’s disease (PD), Lewy body disease (LBD), and frontotemporal dementia (FTD) [13, 14].

The clinical severity of dementia was graded using the Clinical Dementia Rating (CDR), which evaluates cognitive and functional performance in six different areas (memory, orientation, judgment and problem solving, social activities, home and leisure, and personal care), with a score from zero (normal patient) to five (final stage of dementia) [15].

### Functional and cognitive evaluations

All the patients underwent a multidimensional evaluation, conducted by a trained geriatrician. Briefly, the following parameters were assessed: (1) functional status evaluated with Katz’s ADL index [16] and Lawton–Brody index for instrumental ADL (IADL) [17]; (2) cognitive status evaluated using the mini-mental state examination (MMSE) with a score from 0 to 30, adjusted for age and educational level [18]; BPSD, i.e., irritability, apathy, agitation, anxiety, depression, sleep disturbances, aggressiveness, wandering, appetite change, hallucinations, and delusions were assessed using the NPI [19] with a score from 0 to 144; Caregiver’s stress was also evaluated using the NPI with a score from 0 to 60 [19]. NPI was applied only to participants affected by dementia.

### Statistical analysis

Normal distributions of continuous variables were tested using the Kolmogorov–Smirnov test. Data are presented as mean and standard deviation (SD) in case of continuous variables normally distributed or as median in case of non-normal distribution and as frequency and percentage (%) in case of categorical nature of the variables. For descriptive purposes, we present the data in the sample as whole and according to the severity of cognitive impairment, i.e., MCI, dementia in CDR 1–2 (milder forms) and CDR 3–4 (more severe forms). The changes in functional and cognitive status and in NPI (including specific domains) are reported as the difference of post- vs. pre-COVID-19 confinement evaluation. A matched pairwise *t* test was used for analyzing these differences.

Using a logistic regression analysis, we considered the worsening in NPI and in caregiver’s stress (defined as a change between follow-up vs. baseline evaluation > 2 points, corresponding to the median value) as outcomes. All factors associated with a worsening in NPI, or caregiver’s stress (*p* < 0.10) were initially introduced. For selecting the most significant variables associated with the outcomes of interest, a backward logistic regression analysis was applied, leaving only IADL levels at the baseline evaluations and the use of antidepressants during follow-up (this latter only for worsening in NPI). Data of this analysis are reported as odds ratios (ORs) with their 95% confidence intervals (CIs).

All statistical tests were two-tailed, and a *p* value < 0.05 was considered statistically significant. All analyses were performed using SPSS 20.0 software.

## Results

Overall, 100 patients affected by MCI (*n* = 28) or dementia (30 less severe and 42 more severe form according to the CDR) were evaluated before and after COVID-19 quarantine (between January 2019 and May 2021, median follow-up: 10 months). None of the participants reported a diagnosis of COVID-19, during the follow-up period. The mean age of the population was 77.1 years, and 59% of participants were women. Participants were mainly married, living with their families and in the city of Palermo (Table 1). Among the 72 patients affected by dementia, 34 had a diagnosis of AD. Two people had dementia due to PD, two FTD, and one LBD (Table 1). Regarding the functional status, the mean ADL for the whole sample was 4.4/6 ADL and IADL 3.3/8; the mean MMSE was 19.9/30 and, in people affected by dementia, the mean NPI was 14.1 ± 9.2, while the mean caregiver’s stress was 6.1 ± 3.9. Among medications used at the baseline evaluation, antidepressants were the most used (26%), followed by antipsychotics (23%) (Table 1).

**Table 1:** Demographic and clinical characteristics of the patients included at the baseline

Parameter	MCI (*n* = 28)	CDR 1–2 (*n* = 30)	CDR 3–4 (*n* = 42)	All (*n* = 100)
Demographics				
Age (years, mean ± SD)	74.9 (6.7)	76.4 (6.9)	79.0 (6.5)	77.1 (6.8)
Married (*n*, %)	21 (75.0)	18 (60.0)	24 (57.1)	63 (63.0)
Women (*n*, %)	19 (67.9)	15 (50.0)	25 (59.5)	59 (59.0)
Living in city (*n*, %)	25 (89.3)	28 (93.3)	41 (97.6)	94 (94.0)
Living in family (*n*, %)	28 (100)	30 (100)	37 (88.1)	95 (95.0)
Type of dementia				
AD (*n*, %)	–	13 (43.3)	21 (50.0)	34 (34.0)
Mixed (*n*, %)	–	8 (26.7)	12 (28.6)	20 (20.0)
VaD (*n*, %)	–	8 (26.7)	5 (11.9)	13 (13.0)
PD (*n*, %)	–	1 (3.3)	1 (2.4)	2 (2.0)
LBD (*n*, %)	–	0 (0)	1 (2.4)	1 (1.0)
FTD (*n*, %)	–	0	2 (4.8)	2 (2.0)
Functional and cognitive status				
ADL (mean ± SD)	5.4 (1.2)	5.0 (1.0)	3.4 (1.7)	4.4 (1.7)
IADL (mean ± SD)	6.0 (2.1)	3.4 (1.8)	1.5 (1.7)	3.3 (2.6)
MMSE (mean ± SD)	26.4 (2.5)	21.2 (5.3)	14.7 (5.8)	19.9 (6.9)
NPI (mean ± SD)	–	12.3 (9.1)	15.5 (9.2)	14.1 (9.2)
Medications				
Antipsychotics (*n*, %)	2 (7.1)	3(10.0)	18 (42.9)	23 (23.0)
Mood stabilizers (*n*, %)	0 (0)	2 (6.7)	5 (11.9)	7 (7.0)
Anti-Parkinson medications (*n*, %)	1 (3.6)	2 (6.7)	3 (7.1)	6 (6.0)
Benzodiazepines (*n*, %)	2 (7.1)	1 (3.3)	4 (9.5)	7 (7.0)
Antidepressants (*n*, %)	6 (21.4)	13 (43.3)	7 (16.7)	26 (26.0)
Hypnotics (*n*, %)	0 (0)	0 (0)	2 (4.8)	2 (2.0)

*AD* Alzheimer’s disease, *ADL* activities of daily living, *CDR* clinical dementia rating, *FTD* frontotemporal dementia, *IADL* instrumental activities of daily living, *LBD* Lewy body disease, *MCI* mild cognitive impairment, *MMSE* mini-mental state examination, *NPI* neuropsychiatric inventory, *PD* Parkinson’s disease, *VaD* vascular dementia

Table 2 shows the changes in cognitive and functional status before and after COVID-19 quarantine in the sample as a whole and by severity of cognitive impairment. In the sample as a whole, we observed a significant decline in cognitive and functional status after vs. before COVID-19 quarantine: in mean, our participants lost 2.56 points of MMSE and about one ADL and IADL (*p* < 0.0001 for all the comparisons). Regarding cognitive status, from a descriptive point of view, the greatest decline in MMSE was observed in milder forms of dementia (mean change of CDR = 1–2 points and mean change of MMSE = − 3.4 points post- vs. pre-COVID-19 confinement, *p* < 0.0001), followed by more severe forms (mean change of CDR = 3–4 points and mean change of MMSE = − 2.71 points post- vs. pre-COVID-19 confinement, *p* < 0.0001). Similarly, the most evident decline in functional status was observed in milder forms of dementia for both ADL and IADL.

**Table 2:** Change in cognitive and functional status between post- and pre-COVID-19 confinement evaluation

Parameter	All sample (*n* = 100)			MCI (*n* = 28)			CDR 1–2 (*n* = 30)			CDR 3–4 (*n* = 42)		
	Mean difference	SE	*p* value	Mean difference	SE	*p* value	Mean difference	SE	*p* value	Mean difference	SE	*p* value
MMSE	− 2.56	0.39	< 0.0001	− 1.43	0.52	0.01	− 3.40	0.70	< 0.0001	− 2.71	0.68	< 0.0001
ADL	− 0.95	0.12	< 0.0001	− 0.43	0.15	0.008	− 1.23	0.23	< 0.0001	− 1.10	0.20	< 0.0001
IADL	− 0.84	0.13	< 0.0001	− 0.86	0.29	0.006	− 1.03	0.25	< 0.0001	− 0.690	0.18	< 0.0001

*ADL* activities of daily living, *IADL* instrumental activities of daily living, *MMSE* mini-mental state examination, *SE* standard error

Overall, in people affected by dementia, the NPI significantly increased 3.56 ± 8.96 points after COVID-19 quarantine, while the caregivers’ stress increased 1.39 ± 3.46 points between the two evaluations (*p* < 0.0001 for both comparisons). Again, the changes were more evident, in a descriptive way, in people in CDR 1–2 vs. CDR 3–4. Tables 3 and 4 report the changes in specific domains of the NPI and the caregiver’s stress in the sample as a whole and by severity of cognitive impairment. In participants affected by dementia, we observed a significant increase in aggressiveness (mean difference = 0.240 ± 1.21; *p* = 0.05), wandering (mean difference = 0.140 ± 0.682; *p* = 0.043), and disinhibition (mean difference = 0.150 ± 0.73; *p* = 0.043), while the other changes did not reach the statistical significance. When considering the severity of dementia, only the change in aggressiveness in people in CDR 1–2 (mean difference = 0.67 ± 1.18; *p* = 0.004) resulted in a statistical significance, while the other changes did not reach statistical significance (Table 3).

**Table 3:** Change in neuropsychiatric inventory values between post- and pre-COVID-19 confinement evaluation

Domain of the NPI	All sample (*n* = 100)			CDR 1–2 (*n* = 30)			CDR 3–4 (*n* = 42)		
	Mean	Standard deviation	*p* value	Mean	Standard deviation	*p* value	Mean	Standard deviation	*p* value
Delusions	0.130	0.812	0.113	0.233	0.728	0.090	0.048	10.035	0.767
Hallucinations	0.080	0.761	0.296	0.233	0.898	0.165	0.048	0.825	0.710
Aggressiveness	0.240	10.207	0.050	0.667	10.184	0.004	0.071	10.295	0.723
Depression	0.070	10.018	0.493	0.100	10.125	0.630	0.024	10.024	0.881
Anxiety	0.080	0.677	0.240	0.233	0.679	0.070	− 0.071	0.601	0.445
Apathy	− 0.030	10.193	0.802	− 0.067	10.230	0.245	0.143	10.002	0.361
Irritability	0.050	10.313	0.704	0.267	10.388	0.301	0.071	10.404	0.743
Wandering	0.140	0.682	0.043	0.267	0.868	0.103	0.119	0.739	0.303
Sleep	0.150	10.373	0.277	− 0.033	10.586	0.909	0.238	10.527	0.318
Appetite change	0.120	0.820	0.146	0.133	0.900	0.423	0.071	0.808	0.570
Euphoria	0.080	0.419	0.059	0.100	0.548	0.326	0.119	0.453	0.096
Disinhibition	0.150	0.730	0.043	0.200	0.714	0.136	0.214	0.951	0.152
Total score	3.560	8.962	< 0.0001	5.933	7.865	< 0.0001	2.952	10.224	0.07

*CDR* clinical dementia rating

**Table 4:** Change in neuropsychiatric inventory values of caregiver’s stress between post- and pre-COVID-19 confinement evaluation

Domain of the NPI	All sample (*n* = 100)			CDR 1–2 (*n* = 30)			CDR 3–4 (*n* = 42)		
	Mean	Standard deviation	*p* value	Mean	Standard deviation	*p* value	Mean	Standard deviation	*p* value
Delusions	0.150	0.880	0.088	0.092	0.267	0.828	0.151	0.088	0.024
Hallucinations	0.150	0.925	0.093	0.108	0.200	10.031	0.188	0.297	0.214
Aggressiveness	0.200	10.054	0.105	0.061	0.467	0.973	0.178	0.014	0.048
Depression	0.020	0.841	0.084	0.812	0.033	0.669	0.122	0.787	0.071
Anxiety	0.060	0.617	0.062	0.333	0.133	0.629	0.115	0.255	− 0.024
Apathy	0.030	0.223	0.022	0.181	− 0.033	0.615	0.112	0.769	0.167
Irritability	0.080	0.872	0.087	0.361	0.300	10.119	0.204	0.153	0.167
Wandering	0.180	0.821	0.082	0.031	0.067	0.254	0.046	0.161	0.071
Sleep	0.130	10.253	0.125	0.302	0.167	0.986	0.180	0.362	0.262
Change of appetite	0.050	0.297	0.030	0.096	0.167	0.913	0.167	0.326	0.071
Euphoria	0.220	0.917	0.092	0.018	0.071	0.342	0.053	0.183	0.167
Disinhibition	0.120	0.729	0.073	0.103	0.367	0.850	0.155	0.025	0.071
Total score	1.39	3.47	0.35	< 0.0001	2.13	3.170	0.579	0.001	1.31

*CDR* clinical dementia rating, *NPI* neuropsychiatric inventory

Similar results were evident when considering the caregiver’s stress as assessed by the NPI (Table 4). The caregiver’s stress increased 1.39 ± 3.46 points (*p* < 0.0001), being more evident in those with milder forms of dementia. Among the single domains, wandering and euphoria significantly increased between the two evaluations. Considering the single domains, by severity of dementia, in milder forms of dementia, we observed a significant increase in aggressiveness and disinhibition caregiver’s stress (Table 4).

Finally, we investigated which factors were significantly associated with worsening in total NPI and caregiver’s stress, taking the median value of 2 points as outcome. As reported in Table 5, use of antidepressants (OR = 3.53; 95% CI 1.30–9.58; *p* = 0.01) was associated with a higher worsening in NPI values, while higher values of IADL at the baseline were associated with a significant lower worsening in NPI (OR = 0.83; 95% CI 0.70–0.98; *p* = 0.03) and caregiver’s stress (OR = 0.85; 95% CI 0.72–0.99; *p* = 0.04) during the follow-up period.

**Table 5:** Association between worsening in neuropsychiatric inventory values and caregiver’s stress and selected parameters

Parameter	Worsening in NPI^1^		Worsening in caregiver’s stress^1^	
	Odds ratio^2^ (95% CI)	*p* value	Odds ratio^2^ (95% CI)	*p* value
IADL	0.83 (0.70–0.98)	0.03	0.85 (0.72–0.99)	0.04
Use of antidepressants during follow-up	3.53 (1.30–9.58)	0.01	–	

^1^Worsening in NPI and in caregiver’s stress were defined as a change between follow-up and baseline evaluation ≥ 2 points, corresponding to the median value

^2^Data are reported as odds ratios with their 95% confidence intervals. Factors were selected using a two-step approach: (a) all factors associated with a worsening in NPI or caregiver’s stress (*p* < 0.10) were initially introduced (i.e., age, gender, use of anti-Parkinsonian drugs, benzodiazepines, Clinical Dementia Rating scale at the baseline, use of antidepressants during follow-up, instrumental activities of daily living at baseline); (b) a backward logistic regression analysis was applied to select the factors more significantly affected to the outcomes of interest

## Discussion

In the present study, we observed a significant increase in BPSD during the COVID-19 lockdown period associated with an overall reduction of cognitive and functional abilities in patients affected by dementia or MCI.

As widely known, BPSD affect almost all patients with dementia, and are associated with a higher risk of hospitalization, mortality, worsening in quality of life, and increased distress for patients’ caregivers [20, 20]. According to our data, and confirmed by other relevant studies regarding the same topic, a significant increase of incidence and gravity of BPSD appeared driven by COVID-19 quarantine. We can justify our findings with several motivations. The common assumption that loneliness, social isolation, and loss of routine activities could be an important cause of increased anxiety and depression can be a first explanation [22, 23]; second, caregiver’s distress might cause an increase of NPI in patients affected by dementia [24]. In this sense, several studies reported that distressed caregivers tend to use emotion-focused rather than problem-focused coping strategies, which has negative influence on the patient’s NPI [24]. Moreover, the rapid cognitive deterioration during the pandemic, the inability of patients to adapt to new living conditions, and the inability to continue their daily activities may have led to the development of apathy and depression, as confirmed by other investigations [25, 26].

Other works have reported a significant increase in NPI scores during quarantine that was greater in patients with mild dementia than in those with advanced dementia. One explanation could be that persons with milder forms of dementia may have undergone radical changes in their lifestyle habits during lockdown than those with severe dementia, who are generally less active [27]. According to some authors, it is possible that people with mild dementia have a greater awareness of the pandemic and the risks of getting sick, and that this information is likely to cause more concern [26, 27].

In our opinion, the clinical practice provided us another important lesson. A patient suffering from dementia, in absence of drug treatment, may lose about three points on the MMSE scale every year [28]. We observed a significant loss of about 3.4 points in a more limited period, overall indicating that lock down may have accelerated the decline in cognitive performance, particularly in milder forms of dementia (CDR 1–2).

Our study added new and relevant data regarding the potential association between BPSD and disability, in this case reported by the patient’s initial IADL. Indeed, if it was obvious that a patient with greater autonomy was better protected from the sudden worsening of the own pathology, it was not equally obvious that the awareness of the pandemic was not intrinsically a potentially precipitating stress factor. What emerges, in our opinion, is that the more the instrumental skills were preserved, the less worsening the results at the MMSE and the NPI occurred. However, other investigations are needed to confirm our findings.

The findings of our study must be interpreted within its limitations. A first limitation of this study was represented by the large variability of the number of patients per single pathological entity taken in analysis. In many cases, the deferred administration of the NPI for obvious reasons was not administered immediately before and after the start of containment measures. Second, the data only include patients seeking care in one center and only in case of persons affected by dementia/MCI who had urgent need of medical assessment, despite the pandemic. Therefore, a selection bias is possible in our findings (i.e., pre-selection of those who were experiencing and concerned of accelerating decline). Finally, another limitation is the lack of a control group that did not permit to support the idea that a similar population, not in pandemic lockdown, would have had similar losses; therefore, it is hard to know how much of the association is driven by the confinement itself, by social isolation in particular, by the fear of infection, or by the combination of all these possible reasons.

In conclusion, our study indicates that during COVID-19 confinement, a significant decline in functional, cognitive and neuropsychiatric symptoms were present in older people affected by dementia or MCI, particularly in milder forms of dementia. The worsening effect of restrictive measures on persons with mild/moderate dementia could lay the foundations for new strategies and guidelines aimed at managing similar and sudden events of comparable magnitude. Our results reminds us that “Man is a social animal” throughout life. Social relationships contribute in a fundamental way to the development of cognitive functions in the first phase of human life and are the cornerstone on which to base the strategies for maintaining cognitive reserve when aging and pathological processes take place.
